# Supplementary material for: Meta-Analysis and Gene Set Enrichment Relative to ER Status Reveal Elevated Activity of MYC and E2F in the “Basal” Breast Cancer Subgroup
Source: PLoS One. 2009 Mar 9;4(3):e4710. doi: 10.1371/journal.pone.0004710 (PMC2650420; doi:10.1371/journal.pone.0004710)
Supplement: Dataset S1 — GSEA results from first screen. The file contains a zipped archive of the HTML results of the second GSEA screen. Reports for enriched or depleted datasets may be accessed from the “index.html” link within the my_analysis.GseaPreranked.1219975950625.rpt.zip ZIP archive. Enrichment plots are available for each of the 50 most enriched and depleted gene sets. (13.32 MB ZIP) [file pone.0004710.s001.zip › my_analysis.GseaPreranked.1219975950625.rpt.zip!//SHEPARD_CRASH_AND_BURN_MUT_VS_WT_DN.html]

Details for gene set SHEPARD\_CRASH\_AND\_BURN\_MUT\_VS\_WT\_DN[GSEA]

|  || Dataset | zSco.ERneg.to.ERpos\_collapsed |
| Phenotype | NoPhenotypeAvailable |
| Upregulated in class | na\_pos |
| GeneSet | SHEPARD\_CRASH\_AND\_BURN\_MUT\_VS\_WT\_DN |
| Enrichment Score (ES) | 0.5561573 |
| Normalized Enrichment Score (NES) | 2.38549 |
| Nominal p-value | 0.0 |
| FDR q-value | 0.0 |
| FWER p-Value | 0.0 |
Table: GSEA Results Summary

  

Fig 1: Enrichment plot: SHEPARD\_CRASH\_AND\_BURN\_MUT\_VS\_WT\_DN      
 Profile of the Running ES Score & Positions of GeneSet Members on the Rank Ordered List

  

| PROBE | GENE SYMBOL | GENE\_TITLE | RANK IN GENE LIST | RANK METRIC SCORE | RUNNING ES | CORE ENRICHMENT || 1 | CDC20 |  |  | 9 | 7.151 | 0.0200 | Yes |
| 2 | TTK |  |  | 11 | 7.059 | 0.0404 | Yes |
| 3 | FOXM1 |  |  | 21 | 6.760 | 0.0593 | Yes |
| 4 | HDAC2 |  |  | 32 | 6.511 | 0.0774 | Yes |
| 5 | SFRP1 |  |  | 44 | 6.369 | 0.0950 | Yes |
| 6 | AURKB |  |  | 76 | 6.038 | 0.1102 | Yes |
| 7 | MKI67 |  |  | 98 | 5.871 | 0.1256 | Yes |
| 8 | KIF2C |  |  | 124 | 5.703 | 0.1402 | Yes |
| 9 | GTSE1 |  |  | 129 | 5.694 | 0.1564 | Yes |
| 10 | MELK |  |  | 181 | 5.424 | 0.1683 | Yes |
| 11 | DEK |  |  | 190 | 5.364 | 0.1832 | Yes |
| 12 | KIF14 |  |  | 221 | 5.215 | 0.1960 | Yes |
| 13 | BIRC5 |  |  | 229 | 5.191 | 0.2105 | Yes |
| 14 | TPX2 |  |  | 231 | 5.180 | 0.2255 | Yes |
| 15 | PLEKHF1 |  |  | 232 | 5.174 | 0.2405 | Yes |
| 16 | CKS2 |  |  | 239 | 5.145 | 0.2549 | Yes |
| 17 | HMGA1 |  |  | 244 | 5.125 | 0.2694 | Yes |
| 18 | SOX11 |  |  | 250 | 5.094 | 0.2838 | Yes |
| 19 | BUB1B |  |  | 271 | 5.016 | 0.2968 | Yes |
| 20 | CDC14B |  |  | 280 | 4.994 | 0.3107 | Yes |
| 21 | OIP5 |  |  | 320 | 4.868 | 0.3218 | Yes |
| 22 | CDCA3 |  |  | 321 | 4.868 | 0.3360 | Yes |
| 23 | ANP32B |  |  | 329 | 4.860 | 0.3495 | Yes |
| 24 | LMNB2 |  |  | 345 | 4.820 | 0.3623 | Yes |
| 25 | ASPM |  |  | 359 | 4.767 | 0.3751 | Yes |
| 26 | NFIL3 |  |  | 379 | 4.733 | 0.3874 | Yes |
| 27 | SYNCRIP |  |  | 402 | 4.679 | 0.3993 | Yes |
| 28 | CCNB2 |  |  | 431 | 4.589 | 0.4105 | Yes |
| 29 | DLG7 |  |  | 521 | 4.306 | 0.4162 | Yes |
| 30 | CDC27 |  |  | 523 | 4.294 | 0.4286 | Yes |
| 31 | PLK4 |  |  | 524 | 4.292 | 0.4410 | Yes |
| 32 | PRC1 |  |  | 543 | 4.253 | 0.4520 | Yes |
| 33 | MYO10 |  |  | 611 | 4.111 | 0.4588 | Yes |
| 34 | TACC3 |  |  | 669 | 3.995 | 0.4660 | Yes |
| 35 | KIF11 |  |  | 713 | 3.909 | 0.4741 | Yes |
| 36 | FZD9 |  |  | 719 | 3.900 | 0.4850 | Yes |
| 37 | TMPO |  |  | 792 | 3.788 | 0.4905 | Yes |
| 38 | RLF |  |  | 914 | 3.618 | 0.4918 | Yes |
| 39 | PRIM2A |  |  | 941 | 3.564 | 0.5002 | Yes |
| 40 | SHCBP1 |  |  | 986 | 3.501 | 0.5070 | Yes |
| 41 | HMGN3 |  |  | 1005 | 3.478 | 0.5157 | Yes |
| 42 | BMS1L |  |  | 1028 | 3.446 | 0.5240 | Yes |
| 43 | KIF23 |  |  | 1030 | 3.442 | 0.5339 | Yes |
| 44 | CDC2 |  |  | 1259 | 3.182 | 0.5258 | Yes |
| 45 | GUCA1A |  |  | 1295 | 3.145 | 0.5323 | Yes |
| 46 | FBXO5 |  |  | 1304 | 3.137 | 0.5408 | Yes |
| 47 | ATP1A1 |  |  | 1390 | 3.048 | 0.5432 | Yes |
| 48 | CKAP2 |  |  | 1492 | 2.951 | 0.5441 | Yes |
| 49 | CCNB1 |  |  | 1626 | 2.823 | 0.5421 | Yes |
| 50 | NOVA2 |  |  | 1786 | 2.704 | 0.5379 | Yes |
| 51 | ELAVL4 |  |  | 1820 | 2.678 | 0.5432 | Yes |
| 52 | GAS1 |  |  | 1982 | 2.544 | 0.5383 | Yes |
| 53 | FUBP1 |  |  | 2025 | 2.509 | 0.5424 | Yes |
| 54 | ECT2 |  |  | 2026 | 2.509 | 0.5497 | Yes |
| 55 | MYCL1 |  |  | 2037 | 2.497 | 0.5562 | Yes |
| 56 | HLX1 |  |  | 2213 | 2.380 | 0.5498 | No |
| 57 | PCDHGC3 |  |  | 2277 | 2.334 | 0.5518 | No |
| 58 | NDUFB3 |  |  | 2537 | 2.181 | 0.5384 | No |
| 59 | TK1 |  |  | 2730 | 2.076 | 0.5299 | No |
| 60 | PLP1 |  |  | 2842 | 2.019 | 0.5273 | No |
| 61 | FZD5 |  |  | 3099 | 1.887 | 0.5134 | No |
| 62 | TNXB |  |  | 3236 | 1.822 | 0.5083 | No |
| 63 | CTSL |  |  | 3453 | 1.729 | 0.4970 | No |
| 64 | HEY1 |  |  | 3698 | 1.621 | 0.4831 | No |
| 65 | GRIK3 |  |  | 3864 | 1.567 | 0.4752 | No |
| 66 | CRYBB1 |  |  | 4008 | 1.509 | 0.4687 | No |
| 67 | DPYSL3 |  |  | 4053 | 1.491 | 0.4697 | No |
| 68 | RACGAP1 |  |  | 4097 | 1.475 | 0.4707 | No |
| 69 | JAG1 |  |  | 4252 | 1.412 | 0.4631 | No |
| 70 | PHF3 |  |  | 4347 | 1.373 | 0.4599 | No |
| 71 | FOXB1 |  |  | 4403 | 1.355 | 0.4597 | No |
| 72 | MBD2 |  |  | 4505 | 1.315 | 0.4558 | No |
| 73 | ZNF184 |  |  | 4592 | 1.290 | 0.4531 | No |
| 74 | LGMN |  |  | 4615 | 1.281 | 0.4551 | No |
| 75 | KIAA1731 |  |  | 4700 | 1.253 | 0.4524 | No |
| 76 | PUM1 |  |  | 4778 | 1.229 | 0.4501 | No |
| 77 | GFAP |  |  | 4853 | 1.205 | 0.4480 | No |
| 78 | TTN |  |  | 4935 | 1.176 | 0.4452 | No |
| 79 | MYH8 |  |  | 4956 | 1.170 | 0.4471 | No |
| 80 | MT2A |  |  | 5023 | 1.144 | 0.4454 | No |
| 81 | ADARB2 |  |  | 5038 | 1.140 | 0.4476 | No |
| 82 | NOTCH2 |  |  | 5101 | 1.116 | 0.4462 | No |
| 83 | SLIT3 |  |  | 5203 | 1.080 | 0.4416 | No |
| 84 | ZP2 |  |  | 5282 | 1.055 | 0.4388 | No |
| 85 | FLJ12547 |  |  | 5436 | 0.999 | 0.4301 | No |
| 86 | PON1 |  |  | 5679 | 0.921 | 0.4144 | No |
| 87 | POU3F1 |  |  | 5694 | 0.914 | 0.4160 | No |
| 88 | POU3F2 |  |  | 5743 | 0.898 | 0.4149 | No |
| 89 | OLIG2 |  |  | 5815 | 0.873 | 0.4121 | No |
| 90 | KHDRBS1 |  |  | 5819 | 0.872 | 0.4144 | No |
| 91 | EN2 |  |  | 5826 | 0.870 | 0.4164 | No |
| 92 | GDF10 |  |  | 5841 | 0.864 | 0.4179 | No |
| 93 | DIO2 |  |  | 5847 | 0.861 | 0.4200 | No |
| 94 | POU3F3 |  |  | 5942 | 0.834 | 0.4153 | No |
| 95 | SLC34A1 |  |  | 6142 | 0.771 | 0.4024 | No |
| 96 | GREB1 |  |  | 6264 | 0.727 | 0.3953 | No |
| 97 | SOX21 |  |  | 6441 | 0.670 | 0.3839 | No |
| 98 | NEUROG1 |  |  | 6444 | 0.669 | 0.3857 | No |
| 99 | CHD5 |  |  | 6492 | 0.653 | 0.3841 | No |
| 100 | FZD10 |  |  | 6497 | 0.650 | 0.3856 | No |
| 101 | NEUROD4 |  |  | 6550 | 0.634 | 0.3835 | No |
| 102 | G3BP2 |  |  | 6625 | 0.614 | 0.3797 | No |
| 103 | SMTN |  |  | 6675 | 0.598 | 0.3777 | No |
| 104 | ASCL1 |  |  | 6905 | 0.517 | 0.3618 | No |
| 105 | TUBA8 |  |  | 7004 | 0.486 | 0.3558 | No |
| 106 | RHO |  |  | 7363 | 0.361 | 0.3297 | No |
| 107 | ITGB1BP3 |  |  | 7464 | 0.323 | 0.3231 | No |
| 108 | CRYBA4 |  |  | 7654 | 0.246 | 0.3094 | No |
| 109 | PTEN |  |  | 7664 | 0.243 | 0.3095 | No |
| 110 | ENO3 |  |  | 7892 | 0.150 | 0.2927 | No |
| 111 | MDK |  |  | 8042 | 0.089 | 0.2816 | No |
| 112 | AKAP9 |  |  | 8228 | 0.023 | 0.2677 | No |
| 113 | LHX1 |  |  | 8257 | 0.013 | 0.2656 | No |
| 114 | HOXB3 |  |  | 8265 | 0.011 | 0.2651 | No |
| 115 | SOX3 |  |  | 8462 | -0.061 | 0.2504 | No |
| 116 | PTPRN |  |  | 8730 | -0.181 | 0.2307 | No |
| 117 | EDG1 |  |  | 9268 | -0.440 | 0.1912 | No |
| 118 | PAX2 |  |  | 9439 | -0.534 | 0.1799 | No |
| 119 | BUB3 |  |  | 9557 | -0.594 | 0.1727 | No |
| 120 | FGFR4 |  |  | 9832 | -0.745 | 0.1541 | No |
| 121 | DDX17 |  |  | 9961 | -0.821 | 0.1467 | No |
| 122 | PTPRO |  |  | 10017 | -0.857 | 0.1451 | No |
| 123 | ARID5B |  |  | 10415 | -1.097 | 0.1181 | No |
| 124 | NR2F1 |  |  | 10730 | -1.335 | 0.0982 | No |
| 125 | UTX |  |  | 10794 | -1.384 | 0.0974 | No |
| 126 | INSM1 |  |  | 11073 | -1.642 | 0.0811 | No |
| 127 | AP4M1 |  |  | 11123 | -1.683 | 0.0822 | No |
| 128 | ARNT2 |  |  | 11302 | -1.826 | 0.0740 | No |
| 129 | ARHGAP12 |  |  | 11860 | -2.380 | 0.0387 | No |
| 130 | NUMA1 |  |  | 12034 | -2.614 | 0.0331 | No |
| 131 | PPP1R3C |  |  | 12191 | -2.833 | 0.0295 | No |
| 132 | UCP2 |  |  | 12270 | -2.930 | 0.0321 | No |
| 133 | TNRC9 |  |  | 12280 | -2.941 | 0.0399 | No |
| 134 | GOLGB1 |  |  | 12478 | -3.228 | 0.0343 | No |
| 135 | TM7SF2 |  |  | 12522 | -3.295 | 0.0406 | No |
| 136 | NDE1 |  |  | 12586 | -3.408 | 0.0457 | No |
| 137 | NELL2 |  |  | 12593 | -3.420 | 0.0551 | No |
Table: GSEA details [plain text format]

  

Fig 2: SHEPARD\_CRASH\_AND\_BURN\_MUT\_VS\_WT\_DN: Random ES distribution      
 Gene set null distribution of ES for **SHEPARD\_CRASH\_AND\_BURN\_MUT\_VS\_WT\_DN**
